# Are Tree Species Diversity and Genotypic Diversity Effects on Insect Herbivores Mediated by Ants?

**DOI:** 10.1371/journal.pone.0132671

**Published:** 2015-08-04

**Authors:** María José Campos-Navarrete, Luis Abdala-Roberts, Miguel A. Munguía-Rosas, Víctor Parra-Tabla

**Affiliations:** 1 Departamento de Ecología Tropical, Campus de Ciencias Biológicas y Agropecuarias, Universidad Autόnoma de Yucatán, Apartado Postal 4–116, Itzimná, 97000, Mérida, Yucatán, México; 2 Departamento de Ecología Humana, Centro de Investigación y de Estudios Avanzados del Instituto Politécnico Nacional (CINVESTAV), KM 6 Antigua Carretera a Progreso, Mérida, 97310, México; Helmholtz Centre for Environmental Research (UFZ), GERMANY

## Abstract

Plant diversity can influence predators and omnivores and such effects may in turn influence herbivores and plants. However, evidence for these ecological feedbacks is rare. We evaluated if the effects of tree species (SD) and genotypic diversity (GD) on the abundance of different guilds of insect herbivores associated with big-leaf mahogany (*Swietenia macrophylla*) were contingent upon the protective effects of ants tending extra-floral nectaries of this species. This study was conducted within a larger experiment consisting of mahogany monocultures and species polycultures of four species and –within each of these two plot types– mahogany was represented by either one or four maternal families. We selected 24 plots spanning these treatment combinations, 10 mahogany plants/plot, and within each plot experimentally reduced ant abundance on half of the selected plants, and surveyed ant and herbivore abundance. There were positive effects of SD on generalist leaf-chewers and sap-feeders, but for the latter group this effect depended on the ant reduction treatment: SD positively influenced sap-feeders under ambient ant abundance but had no effect when ant abundance was reduced; at the same time, ants had negative effects on sap feeders in monoculture but no effect in polyculture. In contrast, SD did not influence specialist stem-borers or leaf-miners and this effect was not contingent upon ant reduction. Finally, GD did not influence any of the herbivore guilds studied, and such effects did not depend on the ant treatment. Overall, we show that tree species diversity influenced interactions between a focal plant species (mahogany) and ants, and that such effects in turn mediated plant diversity effects on some (sap-feeders) but not all the herbivores guilds studied. Our results suggest that the observed patterns are dependent on the combined effects of herbivore identity, diet breadth, and the source of plant diversity.

## Introduction

Although the effects of plant diversity on primary productivity and plant competitive dynamics have received much attention over the last two decades [1, 2, 3,4], there is also increasing evidence for plant diversity effects on higher trophic levels [3,4,5,6,7,8,9,10].

In particular, a number of studies have shown that both intra- and inter-specific plant diversity, measured as the number of genotypes of a given species or the number of species, respectively influence consumers through increased primary production (greater resource availability) [7,8,11,12,13], as well as through changes in consumer foraging behavior due to increased habitat heterogeneity [14–16].

The effects of plant diversity on insect herbivores have received considerable attention [17–19]. Plant diversity may reduce or increase herbivore abundance, depending on traits such as herbivore dietary specialization [18] as well as the strength of predator top-down effects [3, 4]. With regards to diet breadth, negative effects of diversity are common for specialist herbivores because herbivore recruitment to host plants is frequently density-dependent, and the density of the preferred host plant is lower at high diversity if total plant abundance is held constant, resulting in lower recruitment to diverse patches (“Resource Concentration Hypothesis”) [16, 20]. In contrast, diversity generally does not influence the abundance of generalist herbivores since these are not sensitive to changes in density of any given plant species, and in some cases diversity effects may even be positive due to diet mixing [21,22,23].

Studies have also shown that plant diversity favors greater omnivore or predator (e.g. ants) abundance and diversity because of increased availability of refuges and diversity of resources (“Enemies Hypothesis”) [24, 25], and this is expected to drive greater predation rates and thus reductions in herbivore abundance. The effects of omnivores and predators on herbivores may in turn depend upon herbivore diet breadth [26, 27]. Generalist herbivores are usually more susceptible to predators and parasitoids because they lack mechanisms or traits to avoid predation or parasitism which are frequently found in specialists (e.g. sequestration of plant defenses, crypsis) [26, 28, 29]. In addition, the source of plant diversity (intra- or interspecific) may also modulate predator effects. Because the magnitude of variation in traits is greater among plant species than among maternal families within a species [25], species diversity effects generate greater resource heterogeneity and should thus lead to stronger positive effects on predator abundance and thus enhanced suppression of herbivore populations.

Although the effects of plant diversity on predators have been widely documented [17, 24, 30], most studies have failed to manipulate predator abundance or diversity and thus formally test for predator-mediated diversity effects on herbivores and plants [4, 31]. Recent work suggests that feedbacks between plant diversity and predator effects are an important mechanism by which plant diversity influences herbivory and primary productivity [3]. For example, in a recent study Moreira *et al*. [4] found that positive effects of plant species diversity were contingent upon the presence of predatory ants; diversity effects on plant growth were only observed in the presence of ants and were likely mediated by negative effects of ants on herbivores. In addition, predator effects are expected to vary depending on herbivore diet breadth and the source of plant diversity, but most studies have not accounted for such differences [12, 15]. Clearly, more work is needed in order to assess the prevalence and strength of these feedbacks and their underlying mechanisms.

Ants are a dominant group of arthropods in tropical and subtropical forests, both in terms of biomass and abundance [32]. By acting as predators, omnivores, or mutualists these insects have substantial effects on plant and arthropod communities [33, 34, 35, 36, 37]. In particular, ants frequently act as plant mutualists where ant-plant interactions are mediated by plant-based resources to which ants recruit (e.g. nectar, food bodies) [38, 39, 40] and in turn provide protection against herbivores (reviewed by Rosumek) [41]. Importantly, studies have shown that ants are sensitive to changing habitat conditions [42, 43], including changes in plant diversity [44, 45, 46]. Based on this, ants probably represent a key (but currently understudied) group of plant mutualists mediating plant diversity effects on herbivores [4].

The present study was conducted within the context of a large-scale forest diversity experiment (7.2-ha, 4780 plants) testing for the effects of tree species diversity and mahogany (*Swietenia macrophylla*) genotypic diversity on higher trophic levels. Previous work in this system has shown that plant species diversity reduces the abundance of dietary specialist insect herbivores feeding on mahogany, but does not influence generalist herbivores [47]. Here, we build from this work and investigate whether ants mediate tree species and genotypic diversity effects on specialist and generalist herbivores on mahogany by experimentally reducing ant abundance at both low and high species and genotypic tree diversity. By only sampling mahogany plants, our test of species diversity addresses the influence of tree species neighborhood on herbivores, ants, and protective effects of ants on one component tree species in the system. In contrast, we sampled all mahogany maternal families at low and high genotypic diversity, thus enabling us to perform a conventional test of genotypic diversity effects on consumers associated with this tree species. We predicted that: (*i*) Plant diversity would positively influence ant abundance, negatively influence specialist herbivores, and have no effect or a positive effect on generalist herbivores; (*ii*) Diversity effects on herbivores would be contingent upon ant abundance, but only for generalist herbivores because these are more susceptible to effects of natural enemies relative to specialist herbivores. We expected that the bottom-up effects of resource heterogeneity (e.g. via diet mixing) leading to greater abundance of generalist herbivores in diverse patches would only occur under reduced ant abundances; under ambient (higher) ant abundances, these bottom-up effects would be offset by stronger top-down effects of ants (which recruit more strongly to diverse patches). Negative diversity effects on specialist herbivores (due to resource concentration) will not be contingent upon ants because specialists are less vulnerable to natural enemies. Finally, (*iii*) we predicted that the dynamics described above (i.e. effects on herbivores and ants, and mediation of diversity effects by ants) would be stronger for species diversity than for genotypic diversity effects, because greater plant trait variation underlying species diversity should result in stronger effects on consumer abundance and consumer (ant) top-down effects.

## Methods

### Ethical statement

No permits were necessary for the collection of seeds of the tree species used, and no other requirements were needed for establishing the experimental system or conducting field work, however this activities were supervised by Instituto Nacional de Investigaciones Forestales, Agrícolas y Pecuarias (INIFAP, México).

### Focal tree species and mahogany herbivores

Big-leaf mahogany (*S*. *macrophylla*, Meliaceae), is a self-compatible, long-lived perennial tree distributed from southern México to Bolivia [48]. Plants are monoecious, produce unisexual flowers [49], and fruits are woody capsules containing wind-dispersed seeds [50]. This species also produces extra-floral nectaries (EFN’s) which are located at the base of the leaf blades and is visited by several species of ants [51, 52]. Several of these ant species act as plant mutualists and have been shown to provide defense against herbivores in other species of Meliaceae (*Swietenia* spp. and *Khaya* spp.) [53, 54].

At our field site, big-leaf mahogany is fed upon by several species of generalist insect leaf chewers, namely: chrysomelid beetles (Coleoptera: *Diphaulaca* sp. and *Syphrea* sp.) and orthopterans (Orthoptera: *Aidemona* sp., *Melanoplus* sp., *Philophyllia guttulata*, and *Scudderia mexicana*) [55]. Another abundant group of generalist herbivores are sap-feeders, in particular leafhoppers (Hemiptera: Cicadellidae, *Homalodisca* sp., *Oncometopia* sp. and *Pseudophera* sp.) and membracids (Hemiptera: Membracidae, *Ceresa* spp.) [55], some of which were observed being tended by ants (e.g. *Homalodisca* sp.; L. Abdala-Roberts personal observation). These generalist herbivores have been reported to feed on all tree species in this system [47]. On the other hand, the most important specialist herbivores feeding on this species are *Hypsipyla grandella* Zeller (Lepidoptera: Pyralidae) stem-boring caterpillars and *Phyllocnistis meliacella* Becker (Lepidoptera: Gracillariidae) leaf-mining caterpillars. Both species of specialist herbivores are restricted to feeding on only a few species of Meliaceae and only attacked mahogany in the study system. *Hypsipyla grandella* larvae carve tunnels through the terminal shoots, resulting in deformation of the main stem and reduced growth [56, 57]. *Phyllocnistis meliacella* larvae produce serpentine galleries throughout the leaf surface, and usually one caterpillar is found per leaf (caterpillars rarely move among leaves) [58, 59]. Both specialists feed only on four to five species of Meliaceae, including big-leaf mahogany [58, 59].

In tropical forests of the Yucatan Peninsula (Mexico), big-leaf mahogany co-occurs with five other tree species that are also the subject of this experiment (see experimental design ahead), namely: *Tabebuia rosea* (Bertol.) DC. (Bignonaceae), *Ceiba pentandra* (L.) Gaertn. (Malvaceae), *Enterolobium cyclocarpum* (Jacq.) Griseb. (Fabaceae), *Piscidia piscipula* (L.) Sarg. (Fabaceae), and *Cordia dodecandra* A. DC. (Boraginaceae). These species are long-lived, deciduous, and are distributed from central Mexico to South America [48]. All five of these species produce EFNs and previous studies with some of these tree species has shown that nectaries are visited by ants [60, 61].

### Seed sources

From January 2011 to March 2011, we collected seeds of all species from adult trees located in southern Quintana Roo (Mexico) (S1 File, Table A), and germinated at the INIFAP experimental site in Mococha, Yucatan (Mexico) (21°6’40”N, 89°26’35”W). For all species, we collected seeds from six mother trees (distance among trees ranged from 0.5 to 50 km, depending on the species). In the case of mahogany, maternal seed source distances ranged from 3 to 50 km to assure a high enough degree of unrelatedness among maternal lines [50, 62]. Accordingly, we found significant variation among *S*. *macrophylla* maternal families (mixture of full- and half-sibs) in growth-related traits (e.g. canopy size: 2.5-fold), herbivore resistance (e.g. stem borer attack: 3.8-fold) (S1 File, Table B), and chemical defenses (polyphenolics: five-fold variation 10.44 mg/g to 50.39 mg/g; F_5,50_ = 6.30, P < 0.0001; data from Moreira *et al*., [63].

### Study site and experimental design

In December 2011, we established the tree diversity experiment by planting four month-old seedlings at the INIFAP “Uxmal Experimental Site”, located near Muna, Yucatan (100 km southwest of Mococha; 20°24’44”N, 89°45’13”W). Mean annual precipitation at this site is 1200 mm, and mean annual temperature is 25°C. Plants were fertilized once in January 2012 with N, P, and K (20:30:10), and irrigated with 2 L of water three times per week from January 2012 until June 2012, and from January 2013 to June 2013.

The experiment consisted of 74, 21 by 21-m plots covering an area of 7.2 ha. Each plot contained 64 plants (n = 4780 plants), with 3 x 3 m spacing between plants within plots and 6 x 6 m spacing between plots. The experiment was established on a recently cleared site where vegetation was previously composed mostly of grasses and shrubs, and is presently surrounded by a matrix of secondary tropical forest. Mahogany was planted in 59 out of the total 74 plots, and plots with mahogany were classified into four types: a) mahogany monocultures of one maternal families (12 plots, two replicate plots/maternal family), b) mahogany monocultures of four maternal families (20 plots), c) species polycultures within which all *S*. *macrophylla* saplings were of one maternal family (12 plots, two plots/maternal family), and d) species polycultures within which mahogany plants were represented by four maternal families (15 plots). Treatments of both species and genotypic diversity included equal numbers of individuals of four species and four *S*. *macrophylla* maternal families drawn randomly from pools of six species and six maternal families, respectively. All non-mahogany species were equally represented across polycultures (each species present in six polyculture plots). Likewise, *S*. *macrophylla* maternal families were represented in a similar number of *S*. *macrophylla* monocultures of four maternal families (8–9 plots per maternal family), and also in a similar number of species polycultures where *S*. *macrophylla* plants were of four maternal families (9–10 plots per maternal family). Plots of each treatment combination were randomly interspersed throughout the experimental landscape. For this study, we restricted our sampling to a subset of these 59 plots with mahogany, spanning all treatment combinations (see *Ant reduction treatment* ahead). By sampling only mahogany, our test of species diversity effects addresses the effects inter-specific neighborhood diversity on herbivores, ants, and their interactions associated exclusively with this tree species (rather than overall effects of species diversity on consumers, i.e. across all tree species). The same rationale extends to interpreting the interactive effects between species diversity and the ant treatment. In contrast, by sampling all maternal families at low and high genotypic diversity, we provide an overall test of genotypic diversity effects on ants and herbivores associated with this tree species.

### Description of the ant community

Prior to this experiment, we surveyed ant abundance and species identity by sampling individuals of all tree species in polyculture plots (details regarding methodological procedures are described in S2 File). These data showed no differences in ant abundance (F_4,130_ = 0.76, P = 0.55) or species composition (Pseudo F_5_ = 0.01, P = 0.99) among tree species (S1 File, Table C, S2 File), suggesting that they are equivalent host plants to ants (i.e. ants showed no host-tree species preference). In addition, based on another set of surveys performed exclusively on mahogany, we found that the most abundant ant species on mahogany were *Dorymyrmex bicolor* (Wheeler 1916) (Dolichoderinae) and *Mononomorium cyaneum* (Wheeler 1914) (Myrmicinae), both of which were observed visiting mahogany EFNs [55]. These species are common in managed systems with high productivity, are tolerant to disturbance, and have been shown to be moderately aggressive [64, 65] and members of both genera have been reported to establish mutualistic interactions with hemipterans [36]. Other less abundant ant species found on mahogany were: *Ectatomma tuberculatum* (Olivier 1972) (Ponerinae), *Pseudomyrmex caeciliae* (Forel 1913), *Camponotus planatus* (Roger, 1863) (Formicidae), and *Pseudomyrmex gracillis* (Fabricius 1984) [55] (S1 File, Table D). These last four species have also been reported to visit EFNs of several species of Meliaceae [60].

### Ant reduction treatment

In early July 2013, we selected 24 out of the 59 plots where mahogany was planted, with plot sample sizes allocated in the following way: mahogany monocultures of one maternal family “M-G1” = 6 (one replicate plot per maternal family), monocultures of four maternal families “M-G4” = 6, polycultures with one mahogany maternal family “P-G1” = 6 (one replicate plot per maternal family), and polycultures with four mahogany maternal families “P-G4 = 6. Three of these plots were subsequently excluded and not used in the analyses due to complications with the ant reduction treatment (one M-G1 plot and two P-G1 plots). Within each plot, we randomly selected 10 mahogany plants (N = 240 plants) and experimentally reduced ant abundance on half of these plants by applying a sticky barrier (Vaseline, Productos Químicos “Comercio” S.A. de C.V.) around the main stem, 25 cm above ground-level. The average height of mahogany experimental plants was 2.5 ± 0.75 m (S.E.) (range = max. 3.9 m to min. 1.5 m) at the start of the experiment. The canopy of the experimental plants was not touching with that of neighboring plants in any case. We re-applied the barrier every two weeks throughout the duration of the experiment (July 2013- October 2013).

We conducted three surveys of ant abundance on the selected plants of mahogany throughout the sampling season (on day 5, 35, and 65 after the initial establishment of the ant reduction treatment). During these surveys we did not separate counts by ant species and thus pooled abundances across all species for analyses. We inspected the entire plant, including the main stem, branches, and leaves. To evaluate the effectiveness of this treatment, we conducted a repeated measures generalized linear mixed model which tested for the effects of ant reduction (“A”, fixed, two levels), species diversity (“SD”, fixed, two levels), genotypic diversity (“GD”, fixed, two levels), all two-way interactions, and the three-way interaction; survey and plot were included as random effects. The model used a Poisson error distribution with log link function, and was performed in R version 3.1 [66], using the lme4 package [67]. Results showed that the treatment was effective as ant-reduced plants had a significantly lower (40%) number of ants compared to control (ambient) plants (reduced: 7.7 ± 1.6 ants; control: 12.2 ± 2.2 [mean ± S.E.] ants; F_1, 17_ = 7.95, P = 0.004) (S1 File, Table E), and this effect was not contingent upon the level of SD or GD (S1 File, Table E). The observed magnitude of reduction in ant abundance is similar to the natural range of variation in ant abundance observed on mahogany plants in the experiment (e.g. mahogany maternal families ranged from [mean ± SE] 9.6 ± 2.4 to 13.1 ± 1.5 ants) and thus provided a biologically realistic evaluation of ant effects on herbivores (as opposed to a treatment involving total ant exclusion) [65].

### Surveys of herbivore abundance

Using the same 24 plots and mahogany plants sampled within each plot (see above), we conducted three surveys of herbivore abundance on day 5, 35, and 65 after the initial establishment of the ant reduction treatment. Each plant was visually inspected for five to 10 minutes (depending on plant size), and this consisted in a careful examination of the main stem, branches, and foliage First, we performed counts of leaf-chewers and sap feeders which are more mobile, then we recorded ant abundance, and finally we recorded leafminers and stemborers which are sessile. Although visual inspection may result in underestimates of abundance of small, cryptic insects, most of the herbivore species surveyed are conspicuous and easy to detect and we therefore consider that this method was effective in estimating the abundances of the target herbivore guilds. Generalist herbivores were represented by leaf chewers and sap-feeders, whereas specialists were represented by stem borers (*H*. *grandella*) and leaf miners (*P*. *meliacella*). For both groups of generalists as well as for specialist leaf miners, we recorded the number of individuals (or mines in the case of the leaf miner), whereas for specialists stem borers we counted the number of new attack sites per plant as the caterpillars are concealed inside the stem and direct counts are not feasible. However, the number of new attack sites is correlated with the number of caterpillars per plant [68] and thus can be used as a proxy of abundance. It is worth noting that larvae of both specialist herbivores are internal feeders (inside stems or under the leaf surface) which could make them less susceptible to effects of ants.

### Statistical analyses

First, we used a repeated measures generalized linear mixed model (GLMM) to test for the individual and interactive effects of SD and GD on ant abundance, using only control (ambient ant abundance) plants. Second, we used GLMMs to test for the effects of SD, GD, ant reduction (A), all two-way interactions, and the three-way interaction on the abundance of each herbivore group. All of the above models included survey and plot as random effects, and used a Poisson error distribution and log link function. Whenever a two-way interaction was significant, we used *a posteriori* contrasts to test for pairwise differences among means for a given factor within each level of the other factor [69]. Models were performed in R version 3.1. [66], using the lme4 package [67]. Raw data are available (DOI: 10.5061/dryad.4m897).

## Results

### Effects of tree diversity on ant abundance

**Fig 1:**
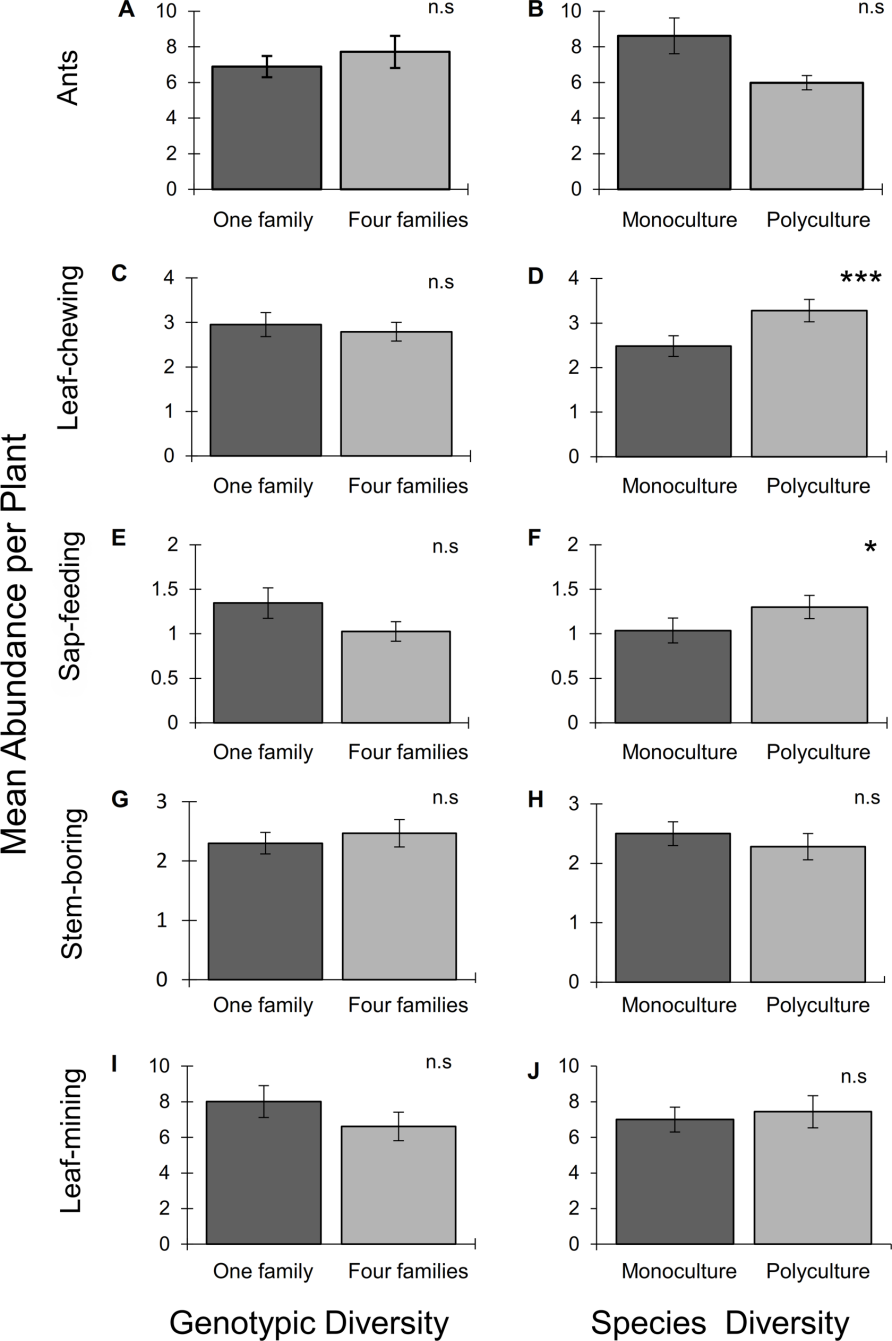
Effects of mahogany (*Swietenia macrophylla*) genotypic diversity and tree species diversity on the abundance of ants (A, B), generalist leaf-chewing herbivores (C, D), generalist sap-feeding herbivores (E, F), specialist stem-boring herbivores (*Hypsipyla grandella*) (G, H), and specialist leaf-mining herbivores (*Phyllocnistis meliacella*) (I, J) on mahogany saplings in a tree diversity experiment in southern Mexico (bars are means ± S.E.). Values are means ± S.E. *P<0.05, **P<0.01, ***P<0.001, n.s. = not significant. G1 = plots with one mahogany maternal family, G4 = plots with four mahogany maternal families; M = monocultures of mahogany, P = polycultures of four species (including mahogany).

We found no effects of SD, GD, or their interaction on ant abundance on mahogany saplings (Table 1; Fig 1A and 1B).

**Table 1:** Results from generalized linear mixed models testing for the effects of an ant reduction treatment (A), tree species diversity (SD), mahogany (*Swietenia macrophylla*) genotypic diversity (GD), and their interactions on the abundance of generalist (G) and specialist (S) herbivores on mahogany. The model for ant abundance tested for the effects of SD, GD, and their interaction, but did not test for the ant reduction treatment or its interactions with SD or GD as it only considered control (ambient ant abundances) plants. All models include survey and plot as random effects. Significant effects (P < 0.05) are in bold.

	Response variable (abundance)				
**Source**	Ants	Leaf chewers (G)	Sap feeders (G)	Stem borers (S)	Leaf miners (S)
**A**	—-	F_1,17_ = 0.09 (0.75)	F_1,17_ = 1.13 (0.28)	F_1,17_ = 0.92 (0.33)	F_1,17_ = 1.23 (0.26)
**GD**	F_1, 17_ = 0.35 (0.58)	F_1, 17_ = 1.21 (0.27)	F_1, 17_ = 1.49 (0.22)	F_1, 17_ = 0.67 (0.41)	F_1,17_ = 0.30 (0.57)
**SD**	F_1,17_ = 1.82 (0.17)	**F**_**1,17**_ **= 9.7 (0.001)**	**F**_**1, 17**_ **= 3.67 (0.05)**	F_1, 17_ = 0.000 (0.99)	F_1, 17_ = 0.16 (0.68)
**SD × A**	—-	F_1,17_ = 0.001 (0.96)	**F**_**1,17**_ **= 5.61 (0.01)**	F_1,17_ = 2.29 (0.08)	F_1,17_ = 1.66 (0.19)
**GD × A**	—-	F_1,17_ = 0.36 (0.54)	F_1,17_ = 0.01 (0.90	F_1,17_ = 1.88 (0.17)	F_1,17_ = 0.51 (0.47)
**SD × GD**	F_1, 17_ = 0.22 (0.63)	F_1,17_ = 0.08 (0.76)	F_1,17_ = 0.34 (0.55)	F_1,17_ = 0.92 (0.33)	F_1,17_ = 0.78 (0.37)
**SD × GD × A**	—-	F_1,17_ = 0.94 (0.33)	F_1,17_ = 1.87 (0.17)	F_1,17_ = 1.29 (0.25)	F_1,17_ = 0.12 (0.72

### Effects of tree diversity and ants on generalist herbivores

We recorded a total of 4141 insects on mahogany throughout the three surveys, of which leaf chewers were the most abundant, followed by ants, and sap feeders (see S1 File, Table F). We found no effect of the ant reduction treatment (A) on the abundance of generalist leaf-chewing herbivores (Table 1). Likewise, there was no effect of mahogany genotypic diversity (GD) on leaf chewers (Table 1; Fig 1C), and this result did not depend on the ant reduction treatment (non-significant GD × A treatment; Table 1). In contrast, there was a significant positive effect of species diversity (SD) on the abundance of leaf chewers (Table 1), with polycultures exhibiting a 33% greater mean abundance than monocultures (Fig 1D). Such effect was not contingent upon the ant reduction treatment (A) (non-significant SD × A interaction; Table 1). In addition, we found no evidence of interactive effects between types of diversity (non-significant SD × GD interaction; Table 1).

**Fig 2:**
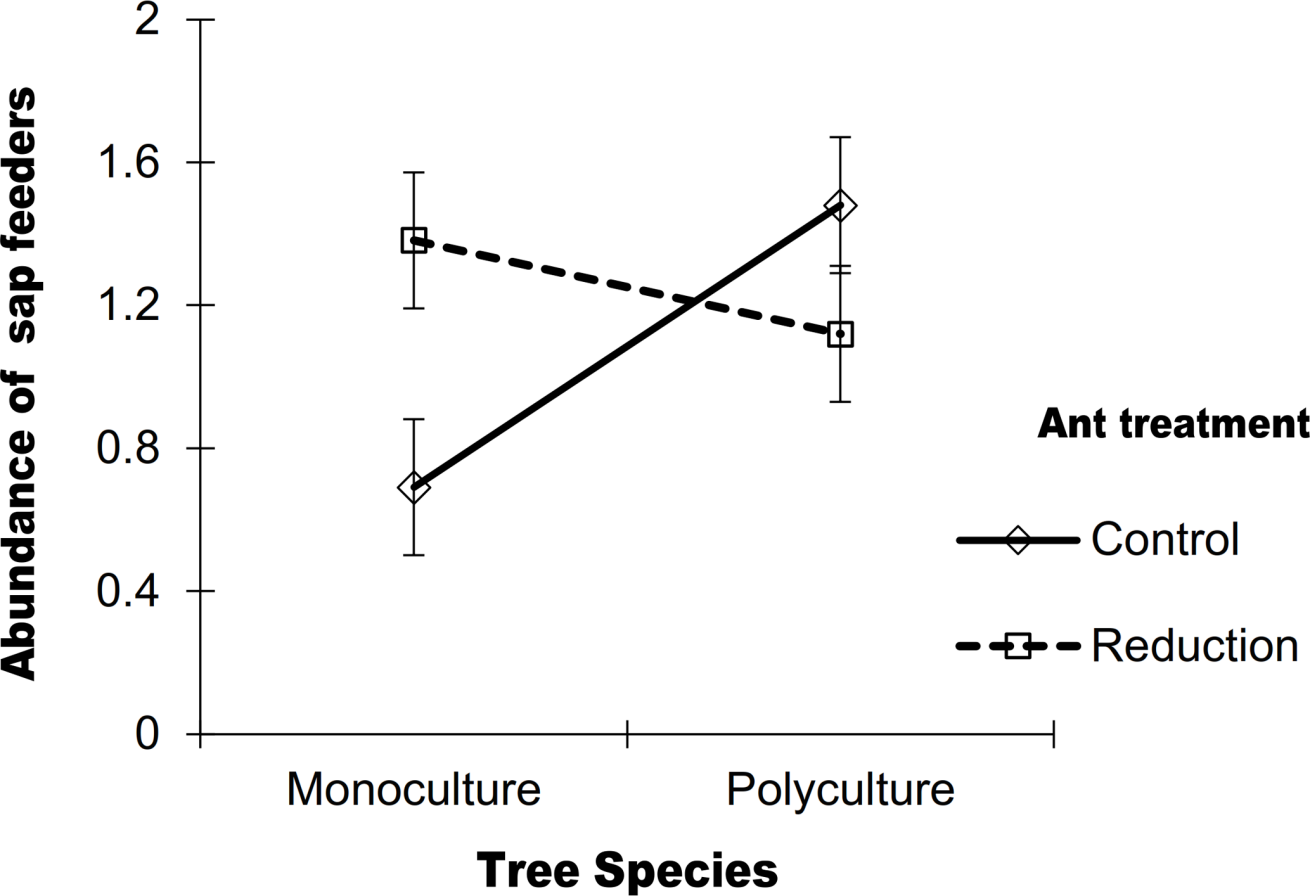
Abundance of sap-feeding generalist herbivores on mahogany (*Swietenia macrophylla*) plants with artificially reduced or ambient (control) ant abundances, across two levels of tree species diversity (mahogany monocultures and polycultures of four tree species, including mahogany), in a tree diversity experiment in southern Mexico. Values are means ± S.E. We found negative effects of ants on sap feeders at low diversity (F_1, 17_ = 5.7, P = 0.01), but not at high diversity (F_1, 17_ = 0.63, P = 0.42), whereas effects of diversity were significant under ambient (control) ant densities (F_1, 17_ = 10.23, P = 0.001) but not under ant-reduced conditions (F_1, 17_ = 1.52, P = 0.21). Statistics are based upon a posteriori contrasts.

There was no overall effect of the ant reduction treatment on generalist sap feeders (Table 1; but see ant treatment × SD interaction ahead). In addition, we did not find a significant effect of GD (Fig 1E), and this effect was not contingent upon the ant reduction treatment (Table 1). In contrast, and as for leaf chewers, we found a significant positive effect of SD on sap feeders (Table 1), with polycultures showing a 20% greater mean abundance than monocultures (Fig 1F). Moreover, in this case SD effects were contingent upon the ant reduction treatment (significant SD × A interaction; Table 1). Specifically, we observed that SD positively influenced sap feeder abundance under ambient ant abundance (50% increase in polyculture relative to monoculture), but had no effect when ant abundance was reduced (Fig 2). At the same time, we found that ants had negative effects on the abundance of sap feeders in mahogany monocultures (sap feeder abundance on ant-reduced plants was greater than on control plants) but not in polycultures (Fig 2). Finally, we found no evidence of interactive effects between types of diversity (Table 1).

### Effects of tree diversity and ants on specialist herbivores

We found no effects of ant reduction, SD, or GD on either specialist stem borers (*H*. *grandella*) or leaf miners (*P*. *meliacella*) (Table 1; Fig 1G–1J). In addition, neither source of plant diversity interacted with the ant reduction treatment (Table 1), and SD and GD effects did not exert interactive effects (Table 1).

## Discussion

### Overview

Tree species diversity had positive effects on the abundance of generalist herbivores (leaf chewers and sap feeders) on mahogany. However, contrary to predictions and in contrast with previous findings in this system [47], specialist herbivores were not influenced by tree species diversity. Likewise, there was no effect of tree species diversity on ant abundance. Despite this latter finding, we found that the effect of tree species diversity on sap feeders was contingent upon ant abundance, with diversity having a positive effect on the abundance of these generalist herbivores group only under ambient (control or non-reduced) ant abundance. Further, we observed a negative effect of ants on sap feeders in mahogany monocultures while in polycultures ants did not influence these herbivores, suggesting that ant effects were influenced by tree species diversity. In contrast, ants did not mediate tree species diversity effects on generalist leaf chewers or specialist herbivores. Finally, mahogany genotypic diversity did not influence herbivore or ant abundance, and ants did not mediate effects of this source of diversity on herbivores.

### Bottom-up effects of tree diversity on herbivores and ants

We found positive effects of tree species diversity on the abundance of generalist insect herbivores feeding on mahogany. Sap feeders and leaf chewers showed 20 to 30% (respectively) higher abundances in polyculture relative to monoculture, which are similar effect sizes to those reported in previous studies for plant diversity effects on generalist insect herbivores [11, 70]. Diversity effects on generalist herbivores are frequently mediated by increased plant biomass at high diversity, resulting in greater herbivore abundance [3, 11]. However, we did not find effects of tree species diversity on mahogany growth [63], which suggests that resource availability did not mediate diversity effects on these herbivores. Alternatively, generalist herbivores could have preferentially recruited to high diversity patches due to a greater chance of finding a preferred host plant [71] or because of positive effects of diet mixing from feeding on multiple plant species [22].

In contrast, we found no effect of tree species diversity on specialist stem-boring (*H*. *grandella*) or leaf-mining (*P*. *meliacella*) caterpillars. This finding contradicts previous studies showing negative responses by specialist herbivores to plant diversity, presumably due to declining host plant density or apparency with increasing diversity at constant plant abundance [72, 73]. In particular, *H*. *grandella* may have overcome reductions in density of mahogany at high diversity because female moths are efficient foragers and have a high dispersal ability [74]. Accordingly, previous work has shown that herbivore mobility is likely an important trait mediating plant diversity effects on herbivores [7, 75]. In addition, current work in this system agrees with these findings as it suggests that mahogany plants are not less apparent (i.e. are not less easy to detect by insect herbivores) in monoculture relative to polyculture. Specifically, these results indicated that the size of focal mahogany plants (mahogany size is correlated with attack by stem borers and leaf miners) [47] was not different to that of neighboring trees in polyculture relative to monoculture (S3 File).

The Enemies Hypothesis predicts greater abundance and diversity of predators at high plant diversity due to greater availability of refuges or resources with increasing diversity [20] reviewed by (Russell) [24]. However, we did not find differences in ant abundance between monocultures and polycultures, thus rejecting the idea that tree species mixtures provide conditions which favored ant recruitment (e.g. increased physical complexity or diversity of plant-based resources) [76]. The dominant ant species associated with mahogany in this system are all common in managed sites and as a result could have been relatively insensitive to changes in abiotic conditions (light availability, humidity) between monocultures and polycultures. In addition, previous surveys indicated that ant abundances were similar for all tree species (i.e. no host-species preference) which could have limited the presence of associational effects (e.g. spill-over of ants from other tree species on mahogany) at high diversity. It is important to note, however, that measurements of ant abundance were conducted relatively early in the establishment of the system (< 2 years). This could be an important consideration in early successional forests dominated by widely-spaced tree saplings, where the effects of habitat heterogeneity on predators take longer to emerge relative to herbaceous systems.

Previous work has found positive effects plant genotypic diversity on arthropod abundance and diversity [8, 11, 13]. In contrast, and despite substantial variation among mahogany maternal families in growth, chemical defenses, and herbivore resistance [47, 63], we did not find effects of genotypic diversity on ants or herbivores. Distance among the sampled mahogany source trees likely represented equal or greater variation than that found at the population level. Consequently, the amount of genotypic variation represented in our experiment probably represented a realistic assessment of genotypic diversity effects by this tree species [47]. Based on this, our results suggest that variation among mahogany maternal families did not offer enough functional contrast to influence consumers (e.g. chemical defenses, architectural complexity, characteristics of EFN nectar in the case of ants) [15]. Correspondingly, mahogany genotypic diversity provided less resource heterogeneity and thus elicited weaker responses by consumers relative to tree species diversity.

### Effects of ants on herbivores and interactive controls of ants and tree diversity

We did not find effects of the ant reduction treatment on most of the surveyed herbivore guilds, except sap feeders (albeit contingent upon species diversity), suggesting that the ant species associated with mahogany in this system do not provide strong protection against herbivores. This result is perhaps not surprising given that ant-plant interactions are very labile and frequently vary in strength and direction depending on the biotic or abiotic context [77]. In this sense, the EFNs of *S*. *macrophylla* are small and not vascularized [51,52], produce lower amounts of nectar compared to other species with larger, vascularized nectaries [78], and likely provide less resources for ants which could explain their weak protective role. In addition, our results are unsupportive of the prediction that predator effects (omnivorous ants in our case) should be stronger on generalist rather than specialist herbivores [27, 28], as generalist leaf chewers were not influenced by the ant reduction treatment and the negative effects of ants on generalist sap suckers were only present in mahogany monocultures indicating that diversity (rather than herbivore diet breadth) mediated top-down effects by ants (see discussion ahead). These results suggest that herbivore diet breadth is not the only trait driving effects of ants and that ant effects may be contingent upon other herbivore traits (e.g. mobility) or habitat characteristics determined by plant diversity.

Importantly, we observed that tree species diversity effects on sap feeders were contingent upon the ant reduction treatment. Specifically, we found that positive effects of diversity on the abundance of these generalist herbivores were only present under ambient ant abundances (control plants), whereas under reduced ant abundance there was no effect of diversity. This result could have been mediated by stronger mutualistic interactions between sap feeders and ants in polyculture relative to monoculture [79]. In this sense, *Homalodisca* sp. leafhoppers and membracids were observed in several cases being tended by ants (L. Abdala-Roberts personal observation). At the same time, however, we found that effects of ants on sap feeders were contingent upon plant diversity; ants had negative effects on these herbivores in mahogany monocultures but had not effect in polycultures (i.e. ant-reduced plants had a higher abundance of sap feeders relative to control plants, but only in monoculture). It is possible that some abiotic feature of diverse environments (e.g. light availability, relative humidity) [80] or habitat physical complexity influenced ant behavior and this resulted in weaker effects on sap feeders at high diversity. In addition, there might have also been diversity effects on ant or herbivore species richness or community composition influencing mahogany-sap feeder-ant interactions, as indicated by previous work in this system showing tree diversity effects on herbivore species richness associated with mahogany [81]. For instance, monocultures may have favored ant species that interact antagonistically (rather than as mutualists) with sap feeders and/or favored sap feeder species that are not tended by ants. Either of these scenarios could have led to the observed decline in abundance of sap feeders in monoculture under ambient ant abundances. Further surveys recording changes in ant and herbivore species identities are needed to test these assertions.

Our findings for ant responses to tree diversity and concomitant effects on herbivores run against predictions from previous work. Whereas prior studies have shown that predator top-down effects strengthen with increasing plant diversity [3, 4], here we show that ant effects are stronger at low diversity. Based on this, we argue that the presence of feedbacks between diversity and predator top-down effects might thus vary as a function of predator traits such as foraging behavior, diet composition (predatory, omnivorous), or preference for particular habitat conditions[82]. Further work in this system will assess diversity effects on abundance and behavior of individual ant and herbivore species, in order to better understand the range of variation in ant-plant-herbivore interactions across levels of tree diversity.

## Conclusions

Our study emphasizes that the effects of plant diversity on herbivores are mediated by predators, omnivores, or other types of plant mutualists. However, predictions on the outcome of these feedbacks will likely depend upon both herbivore and predator traits and how species at each trophic level respond to resource heterogeneity. In addition, findings suggest that the strength and prevalence of such dynamics will probably also depend upon the magnitude (and nature) of plant trait variation underlying different types of diversity (e.g. intra- vs. inter-specific). Based on this, we argue that future work should account for different sources of plant diversity as well as herbivore and predator traits in order to build a more robust theory for predicting the effects of plant-based resource heterogeneity on higher trophic levels and potential feedbacks resulting from such effects.

## Supporting Information

S1 File Including: Table A. Geographical coordinates of trees used as seed sources for the six tree species planted in the forest diversity experiment located in southern México (Yucatán); Table B. General linear models testing for *Swietenia macrophylla* genotypic variation in vegetative traits; Table C. Sample sizes, shown separately for each plot and survey date, for surveys of ant abundance and species richness associated with each plant species in the tree diversity experiment (*Swietenia macrophylla*, *Ceiba pentadra*, *C*. *dodecandra*, *Enterolobium cyclocarpum*, *Piscidia piscipula*, *Tabebuia rosea*); Table D. Total number of individuals recorded for each ant species on big-leaf mahogany (*Swietenia macrophylla*), and percent of the total sample represented by each species; Table E. Results from a generalized linear mixed model testing for the effects of ant reduction treatment (A), species diversity (SD), mahogany (*Swietenia macrophylla*) genotypic diversity (GD), and their interactions on the abundance of ants on mahogany saplings in a tree diversity experiment in southern Mexico; Table F. Total number of ants, insect leaf-chewers, sap-feeders, stem borers (*Hypsipyla grandella*), and leaf miners (*Phyllocnistis meliacella*) recorded on mahogany (*Swietenia macrophylla*) trees during each insect survey date. In the case of stem borers, counts represent the number of new attack sites which is correlated with stem borer abundance;
(DOCX)

S2 File Results from analysis of tree species differences in ant species composition and abundance. (DOCX)

S3 File Results from Plant apparency test. (DOCX)
