# Supplementary material for: Are Tree Species Diversity and Genotypic Diversity Effects on Insect Herbivores Mediated by Ants?
Source: PLoS One. 2015 Aug 4;10(8):e0132671. doi: 10.1371/journal.pone.0132671 (PMC4524695; doi:10.1371/journal.pone.0132671)
Supplement: S1 File — In the case of stem borers, counts represent the number of new attack sites which is correlated with stem borer abundance; (DOCX) [file pone.0132671.s001.docx]

**S1 File. Supplementary material**

## Table A. Geographical coordinates of trees used as seed sources for the six tree species planted in the forest diversity experiment located in southern México (Yucatán).

| Species | Source tree | Coordinates |
| --- | --- | --- |
| *Swietenia macrophylla* | 1 | N334286-E2047987 |
|  | 2 | N334417-E2047202 |
|  | 3 | N353224-E2093310 |
|  | 4 | N349236-E2048027 |
|  | 5 | N355609-E2075999 |
|  | 6 | N357038-E2073319 |
| *Ceiba pentandra* | 1 | N353047-E2065204 |
|  | 2 | N352780-E2064409 |
|  | 3 | N353105-E2065263 |
|  | 4 | N353454-E2065353 |
|  | 5 | N358209-E2047964 |
|  | 6 | N358185-E2047964 |
| *Cordia dodecandra* | 1 | N351419-E2063118 |
|  | 2 | N352337-E2063749 |
|  | 3 | N352346-E2063756 |
|  | 4 | N352188-E2063628 |
|  | 5 | N352437-E2063851 |
|  | 6 | N351095-E2062666 |
| *Enterolobium cyclocarpum* | 1 | N338850-E2074621 |
|  | 2 | N338806-E2074634 |
|  | 3 | N352540-E2064627 |
|  | 4 | N338394-E2074642 |
|  | 5 | N352553-E2064642 |
|  | 6 | N352611-E2064707 |
| *Piscidia piscipula* | 1 | N350889-E2062320 |
|  | 2 | N350883-E2062299 |
|  | 3 | N350874-E2062218 |
|  | 4 | N351043-E2062557 |
|  | 5 | N351090-E2062538 |
|  | 6 | N351012-E2062521 |
| *Tabebuia rosea* | 1 | N357673-E2046984 |
|  | 2 | N357735-E2046970 |
|  | 3 | N356866-E2047303 |
|  | 4 | N357400-E2047064 |
|  | 5 | N357793-E2046956 |
|  | 6 | N356926-E2047276 |

## UTM Zone 16N. Source tree = parental tree from which seeds of each species were sampled.

**Table B. General linear models testing for *Swietenia macrophylla* genotypic variation in vegetative traits.**

| **Response** | **X-fold variation** | **Statistic for maternal family effect** |
| --- | --- | --- |
| Plant height | 1.3 | F_5,731_ = 4.41 **(P = 0.0006)** |
| Number of branches | 1.3 | F_5,731_ = 5.61 **(P < 0.0001)** |
| Canopy volume | 2.6 | F_5,171_ = 2.73 **(P = 0.02)** |
| Stem borer attack | 3.8 | F_5,756_ = 2.58 **(P = 0.02)** |
| Leafminer attack | 1.7 | F_5,184_ = 0.36 (P = 0.87) |
| Leafhopper abundance | 3.1 | F_5,173_ = 1.65 (P = 0.15) |

## This traits was (height, number of branches [both measured in June 2012], canopy volume [October 2012]), and in resistance to *Hypsipyla grandella* stem borers (mean number of new attack sites per plant throughout the rainy season), *Phyllocnistis melliacella* leaf miners (number of leaves with mines per plant, measured in October 2012), and leafhoppers (number per plant, pooled across three species [*Oncometopia* sp. *Peudophera* sp., *Homalodisca* sp.], measured in August and October 2012). All models were performed with PROC MIXED in SAS ver. 9.2 and based upon monoculture plots of *S. macrophylla* of one maternal family using plant as unit of replication and including plot as a random effect. Data were log-transformed to achieve normality of residuals. Shown are F-statistics and P-values, as well as and magnitude of variation between the two most extreme maternal families. Significant effects are in bold. Data from: Abdala-Roberts et al. 2015.

## Table C. Sample sizes, shown separately for each plot and survey date, for surveys of ant abundance and species richness associated with each plant species in the tree diversity experiment (*Swietenia macrophylla, Ceiba pentadra, C. dodecandra, Enterolobium cyclocarpum, Piscidia piscipula, Tabebuia rosea*).

| **Survey** | **# Plot (ID)** | **SD** | **GD** | S. macrophylla | C. pentandra | C. dodecandra | E. cyclocarpum | P. piscipula | | T. rosea |
| --- | --- | --- | --- | --- | --- | --- | --- | --- | --- | --- |
| 1 | 1 | Polyculture | Four families | 2 | 1 | 3 | 1 | 0 | 0 | |
|  | 5 | Polyculture | One family | 1 | 1 | 1 | 1 | 0 | 0 | |
|  | 48 | Polyculture | Four families | 1 | 0 | 0 | 2 | 2 | 1 | |
|  | 56 | Polyculture | One family | 1 | 0 | 1 | 1 | 1 | 0 | |
|  |  |  | **Subtotal** | **5** | **2** | **5** | **5** | **3** | **1** | |
| 2 | 29 | Polyculture | One family | 3 | 8 | 4 | 0 | 0 | 0 | |
|  | 33 | Polyculture | Four families | 3 | 0 | 0 | 2 | 2 | 1 | |
|  | 42 | Polyculture | Four families | 3 | 0 | 6 | 0 | 5 | 5 | |
|  | 69 | Polyculture | One family | 3 | 0 | 0 | 11 | 4 | 4 | |
|  |  |  | **Subtotal** | **12** | **8** | **10** | **13** | **11** | **10** | |
| 3 | 24 | Polyculture | One family | 3 | 6 | 0 | 0 | 6 | 2 | |
|  | 35 | Polyculture | One family | 2 | 5 | 3 | 5 | 0 | 0 | |
|  | 36 | Polyculture | Four families | 3 | 0 | 0 | 1 | 4 | 3 | |
|  | 51 | Polyculture | Four families | 1 | 0 | 0 | 2 | 2 | 1 | |
|  |  |  | **Subtotal** | **9** | **11** | **3** | **8** | **12** | **6** | |
|  |  |  | **Total** | **26** | **21** | **18** | **26** | **26** | **17** | |

## Table D. Total number of individuals recorded for each ant species on big-leaf mahogany (*Swietenia macrophylla*), and percent of the total sample represented by each species.

| Ants species | Abundance | % of simple |
| --- | --- | --- |
| *Dorymyrmex bicolor* | 303 | 77 |
| *Mononomorium cyaneum* | 72 | 18 |
| *Pseudomyrmex caeciliae* | 7 | 2.5 |
| *Ectatomma tuberculatum* | 6 | 1.5 |
| *Camponotus planatus* | 2 | 0.5 |
| *Pseudomyrmex gracillis* | 2 | 0.5 |
| Total | **392** | **100** |

We conducted ant surveys in June 2012, September 2012, and January 2013. During each survey, we randomly selected eight plots of each diversity treatment combination (monocultures of one mahogany maternal family, monocultures of four maternal families, polycultures with one mahogany maternal family, and polycultures with four mahogany maternal families), and within each plot we surveyed 6 to 30 mahogany plants ( N = 467 plants sampled across surveys).

**Table E.** **Results from a generalized linear mixed model testing for the effects of ant reduction treatment (A), species diversity (SD), mahogany (*Swietenia macrophylla*) genotypic diversity (GD), and their interactions on the abundance of ants on mahogany saplings in a tree diversity experiment in southern Mexico.**

| Source of Variation | Effect on ant abundance |
| --- | --- |
| SD | F_1, 17_= 2.83 (0.09) |
| GD | F_1,17_= 0.07 (0.78) |
| A | **F_1,17_= 7.95 (0.004)** |
| SD × GD | F_1, 17=_ 0.0001(0.99) |
| SD × A | F_1, 17=_ 1.07 (0.30) |
| GD × A | F_1, 17=_ 0.44 (0.50) |
| SD × GD × A | F_1, 17=_ 0.73 (0.39) |

This model also included survey and plot as random effects. Analyses were conducted in R ver 3.1, using the lme4 package. We found that the ant reduction treatment significantly reduced ant abundance, and that this effect was consistent across levels of SD and GD. F-values, numerator, denominator degrees of freedom, and P-values (in parenthesis) are shown.

**Table F. Total number of ants, insect leaf-chewers, sap-feeders, stem borers (*Hypsipyla grandella*), and leaf miners (*Phyllocnistis meliacella*) recorded on mahogany (*Swietenia macrophylla*) trees during each insect survey date. In the case of stem borers, counts represent the number of new attack sites which is correlated with stem borer abundance.**

| Survey | Ants | Leaf chewers | Sap feeders | *H. grandella* | *P. meliacella* |
| --- | --- | --- | --- | --- | --- |
| 1 | 476 | 639 | 407 | 64 | 71 |
| 2 | 469 | 358 | 89 | 89 | 149 |
| 3 | 320 | 527 | 105 | 91 | 287 |
| Total | **1265** | **1524** | **601** | **244** | **507** |
