# Supplementary material for: Are Tree Species Diversity and Genotypic Diversity Effects on Insect Herbivores Mediated by Ants?
Source: PLoS One. 2015 Aug 4;10(8):e0132671. doi: 10.1371/journal.pone.0132671 (PMC4524695; doi:10.1371/journal.pone.0132671)
Supplement: S2 File — (DOCX) [file pone.0132671.s002.docx]

## S2 File. Results from analysis of tree species differences in ant species composition and abundance.

## We conducted ant surveys in June 2012, September 2012, and January 2013 on saplings of all tree species located in 12 randomly chosen polyculture plots. Within each plot, we selected multiple plants of each tree species including big-leaf mahogany, *Swietenia macrophylla* (range: 18-26 plants sampled per species, N = 134 total plants sampled). For each plant, we examined the entire canopy and main stem during a five-minute period, counted all ants on the main stem and leaves, and collected specimens for reference collection and identification. Ant specimens were preserved in 70% ethanol and subsequently identified to the lowest possible taxonomic level. To determine if there were tree species differences in ant species composition, we used Permutational Multivariate Analysis of Variance (PERMANOVA) using distance matrices constructed with Bray Curtis index values and 500 permutations. This analysis was performed with the Adonis function in the Vegan package in R version 3.1. Results from this model showed no differences in ant species composition among tree species (Pseudo F5 = 0.01, P = 0.99), indicating that ant community composition was similar across tree species. In addition, we also conducted a generalized linear model to test for differences in overall ant abundance across tree species. This model used a quasi-Poisson distribution to account for overdispersion, and was performed with the GLM function in R version 3.1. Results showed no differences in ant abundance among tree species (F4, 10 =0.76, P =0.55) , suggesting that they are equivalent host plants and this resulted in similar levels of ant recruitment.
