# Supplementary material for: Are Tree Species Diversity and Genotypic Diversity Effects on Insect Herbivores Mediated by Ants?
Source: PLoS One. 2015 Aug 4;10(8):e0132671. doi: 10.1371/journal.pone.0132671 (PMC4524695; doi:10.1371/journal.pone.0132671)
Supplement: S3 File — (DOCX) [file pone.0132671.s003.docx]

**S3 File Results from Plant apparency test.**

**Methods.** *Plant apparency*. We estimated apparency in terms of plant height (proxy of plant size and thus physical interference) by measuring height of the four nearest neighbors of four randomly selected mahogany plants per plot, and calculating an apparency index (AI) in the following way (modified from Castegneyrol et al. 2013):

$$AI= \frac{1}{4}\sum_{i=1}^{4} \frac{HF-{HN}_{i}}{dFN_{i}}$$

Here *HF* is the height of a focal mahogany plant, *HN* is the height of a given neighbor, and *d* is the distance between the focal plant (*F*) and each neighboring plant (*N*). This index estimates the mean difference in heights between focal and neighboring trees, weighted by the distance between the focal tree and it neighbors, resulting in one value per focal tree. Negative AI values indicate that the focal tree was, on average, smaller (i.e. less apparent) than its neighbors (Castagneyrol et al. 2013a). We chose plant height given that this trait is predictive of stem borer attack (e.g. Mo et al. 1997; data from this study). We used a general linear model in PROC GLM to test for an effect of tree species diversity on apparency by comparing mahogany monocultures and species polycultures (irrespective of the level of genotypic diversity).

**Results**. *Plant apparency*. Although there was a tendency for mahogany apparency (AI) to be lower at high species diversity (-0.074 ± 0.03) relative to low species diversity (-0.002 ± 0.02), the difference was not significant (F_1,57_ = 3.09, P = 0.09), thus offering weak support for the expectation that apparency acted as a mechanism of species diversity effects on herbivores associated with mahogany. (Data from Abdala-Roberts et al. 2015)
